# Association of IGF-1 and IGFBP-3 with metabolic abnormalities among children and adolescents

**DOI:** 10.3389/fendo.2025.1579107

**Published:** 2025-06-05

**Authors:** Zhenghao Zhao, Yuanyuan Ma, Xinyi Zhang, Xiaoxiao Liu, Yang Li, Zhongze Fang, Rongxiu Zheng, Jing Li

**Affiliations:** ^1^ Department of Toxicology and Health Inspection and Quarantine, School of Public Health, Tianjin Medical University, Tianjin, China; ^2^ Department of Pediatrics, Tianjin Medical University, General Hospital, Tianjin, China; ^3^ Tianjin Key Laboratory of Environment, Nutrition and Public Health, Tianjin, China; ^4^ Tianjin Center for International Collaborative Research on Environment, Nutrition and Public Health, Tianjin, China; ^5^ Department of Epidemiology and Biostatistics. School of Public Health, Tianjin Medical University, Tianjin, China

**Keywords:** insulin-like growth factor-1, insulin-like growth factor binding protein-3, adolescent, children, metabolic abnormalities

## Abstract

**Background and Objective:**

Insulin-like growth factor-1 (IGF-1) and insulin-like growth factor binding protein-3 (IGFBP-3) play roles in growth and development, but their association with metabolic abnormalities in children and adolescents remains unclear. This study aimed to investigate the relationship between IGF-1, IGFBP-3, and metabolic abnormalities in Chinese children and adolescents, while assessing the role of age in these associations.

**Methods:**

Participants were categorized into low-risk and high-risk groups based on metabolic abnormality criteria. Demographic, anthropometric, and laboratory data were collected via medical records. Logistic regression was used to calculate odds ratios (ORs) and 95% confidence intervals (CIs).

**Results:**

Data from 588 participants were analyzed. Higher IGF-1 (Q4: OR 0.24, 95% CI: 0.11–0.51) and IGFBP-3 levels (Q4: OR 0.38, 95% CI: 0.18–0.76) were associated with lower odds of metabolic abnormalities. Higher IGF-1/IGFBP-3 ratios also reduced metabolic abnormality risk. Age-related trends showed IGF-1 levels plateaued with age, while IGFBP-3 progressively increased, with the low-risk group consistently maintaining higher levels.

**Conclusions:**

Higher IGF-1 and IGFBP-3 levels are negatively associated with metabolic abnormalities in children and adolescents. Maintaining the balance of these factors is critical for metabolic health, especially during adolescence.

## Introduction

The increasing prevalence of obesity is leading to more cases of metabolic abnormalities in youth, posing a major public health concern (1–3). In 2022, among children and adolescents aged 5-19, more than 390 million were overweight, including 160 million with obesity (4). While obesity was once considered a concern primarily for high-income countries, its prevalence is now rising rapidly in low- and middle-income nations (5). Moreover, common metabolic risk indicators such as blood pressure (6), hypertriglyceridemia (7), and low-density lipoprotein cholesterol disorders (8) are increasingly showing abnormal trends among children and adolescents. Therefore, identifying modifiable risk factors in children and adolescents is crucial for improving their metabolic health.

Insulin-like Growth Factor-1(IGF-1) is primarily secreted by the liver under the stimulation of growth hormone (GH) (9, 10) while Insulin-like Growth Factor Binding Protein-3(IGFBP-3) serves as the main binding protein for IGF-1, regulating its biological activity (11). The development of a stable complex between IGFBP-3 and IGF-1 regulates IGF-1 availability and half-life (12, 13) *in vivo*, regulating receptor activation and subsequent signaling pathways (14).

Abnormalities in the IGF-1 axis may be associated with the development of obesity, which in turn may affect metabolic health (15). Research has demonstrated a strong association of IGF-1 and its binding proteins (including GHR and IGFBP-3) in adipose tissue cells with metabolic dysfunction among obese children (16). Furthermore, there are substantial association of IGF-1 and its binding proteins (IGFBP-1, IGFBP-2, and IGFBP-3) with cardiovascular disease risk in children aged 7 to 9 (17). Low IGF-1 is linked to insulin resistance and related metabolic disorders (18). Moreover, lower IGF-1 is associated with the risk of cardiovascular disease, such as hypertension and vascular dysfunction (19). Evidence indicates that the WFS1 gene plays a pivotal role in modulating the insulin-like growth factor-1 (IGF-1) and growth hormone (GH) signaling pathway, with genetic perturbations predisposing to impaired somatic growth and metabolic disturbances, including diabetes mellitus (20, 21). Intriguingly, WFS1 was first characterized in conditioned fear paradigms, linking it to emotional and behavioral regulation (22) and suggesting a mechanistic bridge between metabolic control and affective processes via WFS1mediated regulation of IGF-1/GH activity. IGFBP-3 is associated with all five elements of the metabolic syndrome among the older population (23). Other studies have shown that children who are overweight or obese have higher serum IGFBP-3 levels and that IGFBP-3 concentrations are linked to several cardiovascular risk factors, including obesity and insulin levels (16) (24),. Most of these studies have focused on older populations or metabolic diseases, and no studies have explored the association of IGF-1 and IGBP-3 with metabolic abnormalities assessed by clinical parameters among children and adolescents.

In this cross-sectional study, we aimed to 1) assess the association of IGF-1 and IGFBP-3 and their ratios with metabolic abnormality in adolescents; and 2) explore the trends of IGF-1 and IGFBP-3 with age.

## Methods

### Exclusion criteria

The exclusion criteria for our study were: 1) children who had previously suffered from kidney disease, liver failure, cancer, or other severe systemic diseases; 2) children with hypothalamic diseases, pituitary disorders, thyroid dysfunctions, diabetes, chromosomal abnormalities, or various syndromes; 3) children who had a history of smoking, long-term alcohol consumption, or use of medications that could affect lipid metabolism, blood pressure, liver function, insulin action, blood glucose levels, or body weight.

### Study populations

**Figure 1:**
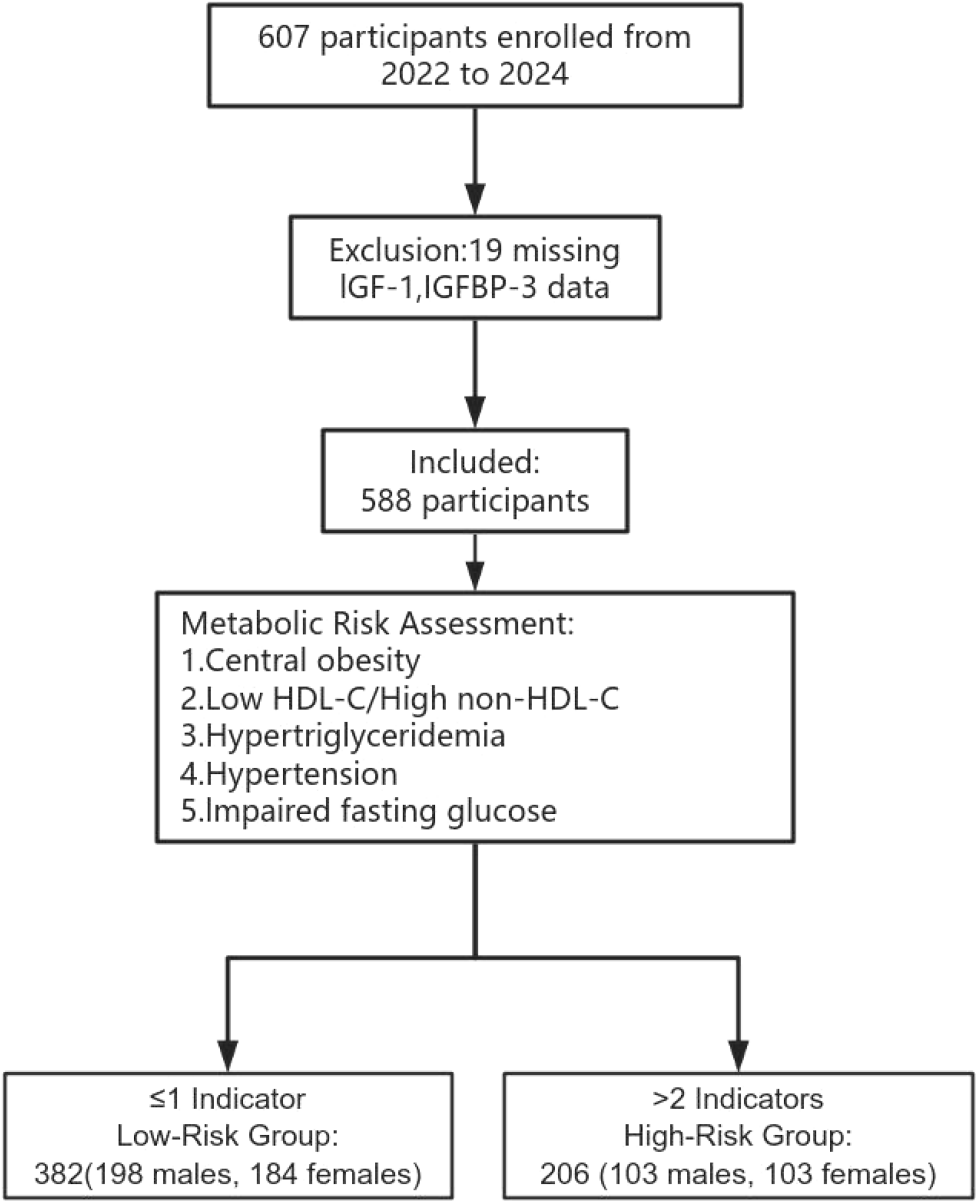
Flowchart of the study population.

This cross-sectional study was conducted between January 2022 and March 2024 at the Department of Pediatrics, Tianjin Medical University General Hospital, Tianjin, China. From January 2022 to March 2023, a total of 607 children were consecutively admitted to the Department of Pediatrics at Tianjin Medical University General Hospital in Tianjin, China, and agreed to participate in this study. Among them, 19 participants were excluded due to missing IGF-1 and IGFBP-3 data. Finally, A total of 588 participants aged 6–17 years were ultimately included in the study (**Figure 1**).

### Measurement of IGF-1 and IGFBP-3

Serum levels of Insulin-like Growth Factor-1 (IGF-1) and Insulin-like Growth Factor Binding Protein-3 (IGFBP-3) were measured using standardized enzyme-linked immunosorbent assay (ELISA) kits. Blood samples were collected from participants after an overnight fast and immediately processed. The serum was separated by centrifugation and stored at -80°C until analysis. The assays were performed in accordance with the manufacturer’s instructions, and each sample was measured in duplicate to ensure accuracy. The concentrations of IGF-1 and IGFBP-3 were reported in nanograms per milliliter (ng/mL). Quality control procedures were followed to minimize variability, and intra- and inter-assay coefficients of variation were maintained within acceptable ranges.

### Assessment of metabolic risk

The diagnosis of metabolic abnormalities is based on the criteria for metabolic syndrome in Chinese children and adolescents proposed by the Pediatric Branch of the Chinese Medical Association in 2012 (25), which includes: 1. Central obesity. 2. Low high-density lipoprotein cholesterol (HDL-C) or high non-high-density lipoprotein cholesterol (non-HDL-C).3. Hypertriglyceridemia. 4. Hypertension. 5. Impaired fasting glucose. Central obesity is diagnosed using waist circumference percentile cutoffs for Chinese children and adolescents aged 7 to 18 years. A low HDL-C is defined as HDL-C < 1.03 mmol/L, or high non-HDL-C as non-HDL-C ≥ 3.76 mmol/L. Hypertriglyceridemia is defined as triglycerides (TG) ≥ 1.47 mmol/L. Hypertension is defined as having a systolic blood pressure (SBP) and/or diastolic blood pressure (DBP) ≥ the 95th percentile for age and sex. Impaired fasting glucose is defined as fasting blood glucose ≥ 5.6 mmol/L. Low-risk (≤1) and high-risk (≥2) groups were categorized based on the number of metabolic risks.

### Data collection

This study’s covariates included anthropometric measurements and data collected through questionnaires. Anthropometric measurements for each participant included weight, height, systolic blood pressure (SBP), diastolic blood pressure (DBP), waist circumference, and hip circumference. All measurements were conducted with participants wearing light clothing and without shoes. Weight was recorded to the nearest 0.1 kilogram, and height was measured to the nearest 0.1 centimeter in the morning. Obesity was assessed using the Body Mass Index (BMI), which was calculated as weight in kilograms divided by height in meters squared (kg/m²). Blood pressure was measured three times after participants had rested in a seated position for 5 minutes, and the average value was used. The definitions of overweight and obesity are based on the “Classification Criteria for Overweight and Obesity Screening in School-age Children and Adolescents” (WS/T 586-2018), which applies to children and adolescents aged 6 to 18 years. Overweight is defined as a BMI between the 85th and 95th percentiles for the same age and gender, while obesity is defined as a BMI at or above the 95th percentile (26).

Waist circumference was measured with participants standing, feet shoulder-width apart. A non-elastic measuring tape was placed around the waist at the narrowest part of the torso, or midway between the iliac crest and the lower rib margin. The tape was kept flat against the skin (or clothing) but not so tight as to compress the skin. Measurements were taken at the end of a natural exhalation and recorded to the nearest 0.1 centimeter. Hip circumference was similarly measured while standing, with feet shoulder-width apart. The tape measure was positioned around the fullest part of the hips, generally just above the upper thighs. The tape was ensured to be level around the body, and measurements were taken at the end of a natural exhalation, and recorded to the nearest 0.1 centimeter. All measurements were performed by trained personnel under standardized conditions to ensure reliability. Multiple measurements were taken, and the average was calculated. The waist-to-hip ratio was determined by dividing waist circumference by hip circumference, expressed as Waist-Hip Ratio (WHR)= waist circumference (cm)/hip circumference (cm).

Data collection also included questionnaires completed by parents at registration. Parents provided information on the family’s socioeconomic status and personal details for both parents, including lifestyle factors such as smoking and drinking. The questionnaire also included questions on the child’s lifestyle habits, sleep patterns, dietary habits, and physical activity. Physical activity was defined as engaging in exercise at least three times a week for more than 30 minutes per session; participants not meeting this criterion were considered not to engage in regular physical activity. Biochemical blood tests were conducted in a specialized diagnostic laboratory using a fully automated biochemical analyzer (Hitachi 7150, Tokyo, Japan). Fasting venous blood samples were collected from each participant between 8:00 AM and 9:30 AM after an 8-hour fasting period. The biochemical examination information of the participants included alanine Aminotransferase (ALT), aspartate aminotransferase (AST), triglycerides (TG), high-density lipoprotein cholesterol (HDL-C), low-density lipoprotein cholesterol (LDL-C), total bilirubin (TBIL), direct bilirubin (DBIL), fasting glucose, fasting insulin, and other related parameters.

### Statistical analysis

The chi-square test for categorical variables and t-test for continuous variables were used to compare the characteristics of the study participants based on metabolic risk.

The odds ratio (OR) and 95% confidence interval (CI) for the associations of IGF-1, IGFBP-3, and IGF-1/IGFBP-3 (continuous and quartile) with metabolic risk were estimated using logistic regression models. In the multivariable models, we adjusted for age, sex, BMI, WHR, ALT, AST, TBIL, DBIL, HOMA, TG, HDL-C, and LDL. The relationships of IGF-1, IGFBP-3, and their ratio with metabolic risk were visualized using the local estimation scatterplot smoothing (LOESS) technique.

In sensitivity analysis, we further evaluated the association between IGF-1, IGFBP-3, and their ratio with metabolic risk by dividing them into tertiles. Additionally, we further adjusted the household income and parental education level.

Two-tailed *P*-values <0.05 were considered statistically significant. All statistical analyses were performed using R Version 4.3.2.

## Results

### The characteristics of the study population

Of the 588 participants included in the analysis (mean [SD]: 11.01 years; 51.19% of female). Compared to the low-risk participants, the high-risk participants had higher age (11.4 ± 2.3 years vs. 10.8 ± 2.2 years), height (153.7 ± 15.9 cm vs. 145.9 ± 15.2 cm), weight (61.1 ± 22.8 kg vs. 42.3 ± 16.4 kg), BMI (25.0 ± 6.27 kg/m² vs. 19.2 ± 4.59 kg/m²), additionally, WHR (0.86 [0.82, 0.89] vs. 0.85 [0.81, 0.88], p = 0.002) and TG (1.51 mmol/L [1.04, 1.91] vs. 0.91 mmol/L [0.67, 1.20], p < 0.001) were also higher in the high-risk group, and HOMA-IR (3.962 [2.418, 6.359] vs. 2.129 [1.321, 3.472], p < 0.001), were also significantly elevated in the high-risk group. Furthermore, ALT (19 U/L (13, 32) vs. 15 U/L (12, 20), p < 0.001) was higher in the high-risk group, while TBIL (8.8 µmol/L [6.9, 12.2] vs. 9.9 µmol/L [7.7, 13.4], p = 0.032) and DBIL (2.7 µmol/L [2.1, 3.6] vs. 3.0 µmol/L [2.2, 4.2], p = 0.004) were lower. (**Table 1** **).**

**Table 1:** The characteristics of the study population among children and adolescents (n = 588).

Characteristics	Low metabolic risk (n = 382)	High metabolic risk (n = 206)	*P*-Value
Age, y	10.8 (2.2)	11.4 (2.3)	0.002
Female	198 (51.83)	103 (50.00)	0.736
BMI (kg/m^2^)	19.2 (4.59)	25.0 (6.27)	<0.001
Normal	319 (83.51)	127 (61.65)	<0.001
Overweight	23 (6.02)	15 (7.28)	
Obesity	40 (10.47)	64 (31.07)	
SBP (mmHg)	106 (99, 112)	120 (109, 128)	<0.001
DBP (mmHg)	67 (63,72)	75 (69, 81)	<0.001
WHR	0.85 (0.81, 0.88)	0.86 (0.82, 0.89)	0.002
TG (mmol/l)	0.91 (0.67, 1.20)	1.51 (1.04, 1.91)	<0.001
HDL-C (mmol/l)	1.30 (1.14, 1.55)	1.09 (0.96, 1.27)	<0.001
Non-HDL-C (mmol/l)	2.91 (2.48, 3.38)	3.23 (2.69, 3.82)	<0.001
LDL-C (mmol/l)	2.35 (1.97, 2.76)	2.61 (2.21, 3.15)	<0.001
FBG (mmol/L)	4.81 (4.54, 5.05)	4.87 (4.58, 5.21)	0.031
Insulin (mU/L)	9.9 (6.3, 15.7)	18.1 (11.0, 28.8)	<0.001
HOMA-IR	2.129 (1.321, 3.472)	3.962 (2.418, 6.359)	<0.001
ALT (U/L)	15 (12, 20)	19 (13, 32)	<0.001
AST (U/L)	22 (19, 27)	22 (17, 29)	0.866
TBIL (umol/L)	9.9 (7.7, 13.4)	8.8 (6.9, 12.2)	0.032
DBIL (umol/L)	3.0 (2.2, 4.2)	2.7 (2.1, 3.6)	0.004
IGF-1 (ng/ml)	334.5 (235.30, 458.50)	276.0 (194.00, 391.80)	0.001
IGFBP-3 (ug/ml)	5.82 (4.99, 6.75)	5.45 (4.59, 6.29)	0.003

Data are presented as mean ± standard deviations, median (interquartile range), or n (%).

BMI, Body Mass Index; SBP, Systolic Blood Pressure; DBP, Diastolic Blood Pressure; AO, Abdominal obesity; IGF-1, Insulin-like Growth Factor-1; IGFBP-3, Insulin-like Growth Factor Binding Protein-3; TC, Total Cholesterol; TG, Triglycerides; HDL-C, High-Density Lipoprotein Cholesterol; Non-HDL-C, Non-High-Density Lipoprotein Cholesterol; LDL-C, Low-Density Lipoprotein Cholesterol; FBG, Fasting Blood Glucose; Insulin, Fasting Insulin; HOMA-IR, Homeostatic Model Assessment of Insulin Resistance; ALT, Alanine Transaminase; AST, Aspartate Transaminase; TBIL, Total Bilirubin; DBIL, Direct Bilirubin.

### Association of IGF and IGFBP-3 with metabolic abnormalities

In the multivariable model, compared to the lowest quartile of IGF-1 and IGFBP-3, the highest quartile of IGF-1 (OR: 0.24, 95% CI: 0.11 to 0.51) and IGFBP-3 (OR: 0.38, 95% CI: 0.18 to 0.76) were negatively associated with metabolic abnormalities (**Table 2**). In the multivariable model, compared with the lowest quartile, Q3 (OR: 0.43; 95% CI: 0.21 to 0.86) and Q4 (OR: 0.47, 95% CI: 0.22 to 0.96) levels of IGF-1/IGFBP-3 were negatively associated with metabolic abnormalities (**Table 3**).Age- and sex-stratified subgroup analyses similarly revealed the same trends(**Supplementary Table S1**, **Supplementary Table S2**).

**Table 2:** The association of IGF-1 and IGFBP-3 with metabolic abnormalities among children and adolescents.

	OR (95% CI)	*P*-value
IGF-1 (ng/ml)		
Model 1		
Q1	Reference	
Q2	0.55 (0.34, 0.89)	0.014
Q3	0.62 (0.38, 0.99)	0.045
Q4	0.39 (0.23, 0.63)	<0.001
Model 2		
Q1	Reference	
Q2	0.50 (0.26, 0.96)	0.038
Q3	0.37 (0.19, 0.74)	0.005
Q4	0.24 (0.12, 0.49)	<0.001
Model 3		
Q1	Reference	
Q2	0.52 (0.26, 1.06)	0.073
Q3	0.33 (0.16, 0.68)	0.003
Q4	0.24 (0.11, 0.51)	<0.001
IGFBP-3 (μg/ml)		
Model 1		
Q1	Reference	
Q2	0.65 (0.41, 1.04)	0.074
Q3	0.59 (0.37, 0.95)	0.031
Q4	0.53 (0.32, 0.85)	0.009
Model 2		
Q1	Reference	
Q2	0.56 (0.29, 1.06)	0.076
Q3	0.46 (0.24, 0.88)	0.020
Q4	0.42 (0.21, 0.83)	0.013
Model 3		
Q1	Reference	
Q2	0.50 (0.25, 1.00)	0.051
Q3	0.42 (0.21, 0.82)	0.013
Q4	0.38 (0.18, 0.76)	0.007

IGF-1: Q1:< 214.8 ng/ml; Q2: 214.8-433.5 ng/ml; Q3: 433.5-983.00 ng/ml; Q4: ≥ 983.00 ng/ml.

IGFBP-3: Q1: < 4.84 μg/ml; Q2: 4.84-5.76 μg/ml; Q3: 5.76-12.00 μg/ml; Q4: ≥ 12.00 μg/ml.

Model 1 is the un-variable model.

Model 2 adjusted for age, sex, BMI, WHR, ALT, AST, TBIL, DBIL, TG, and HOMA.

Model 3 was further adjusted for HDL-C and LDL-C based on Model 2.

**Table 3:** The association between IGF-1/IGFBP-3 ratio and metabolic abnormalities among children and adolescents.

	OR (95% CI)	*P*-value
Model 1		
Q1	Reference	
Q2	0.62 (0.38, 0.99)	0.045
Q3	0.53 (0.32, 0.85)	0.009
Q4	0.56 (0.35, 0.90)	0.018
Model 2		
Q1	Reference	
Q2	0.72 (0.37, 1.39)	0.328
Q3	0.45 (0.24, 0.87)	0.017
Q4	0.45 (0.22, 0.88)	0.020
Model 3		
Q1	Reference	
Q2	0.76 (0.38, 1.54)	0.451
Q3	0.43 (0.21, 0.86)	0.018
Q4	0.47 (0.22, 0.96)	0.040

IGF-1/IGFBP-3 ratio: Q1:<41.165; Q2: 41.165-55.963; Q3: 55.963-73.337; Q4: ≥73.337.

Model 1 is the un-variable model.

Model 2 adjusted for age, sex, BMI, WHR, ALT, AST, TBIL, DBIL, TG, and HOMA.

Model 3 was further adjusted for HDL-C and LDL-C based on Model 2.

### Trends in IGF-1 and IGFBP-3 with age

**Figure 2:**
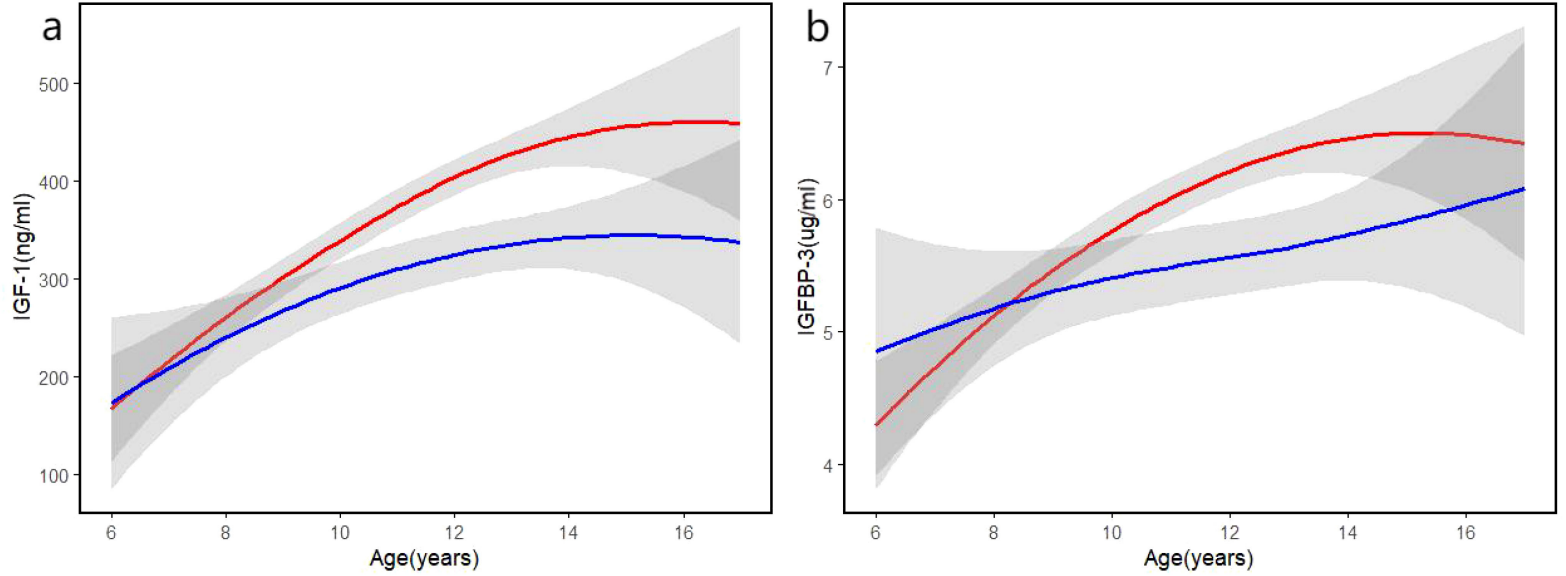
**(a)** Bivariate fit curve of serum IGF-1 concentration with age. **(b)** Bivariate fit curve of serum IGFBP-3 concentration with age. Red line: low risk group; blue line: high risk group. Shaded areas indicate 95% confidence interval.

In both the low-risk and high-risk groups, IGF-1 levels initially increase with age and then plateau. In the low-risk group, IGF-1 levels rise steadily until approximately 12 to 14 years of age, after which they stabilize, while IGF-1 levels in the high-risk group consistently remain lower than those in the low-risk group and also stabilize around 14 years of age. During mid-puberty (12 to 14 years), changes in IGF-1 levels in both groups are relatively stable, whereas greater variability is observed in the early and late stages of puberty (**Figure 2a**). IGFBP-3 levels progressively increase with age in both the low-risk and high-risk groups. Across all age ranges, IGFBP-3 levels are consistently higher in the low-risk group compared to the high-risk group, with the differences becoming particularly pronounced between the ages of 12 and 15 years. After age 15, IGFBP-3 levels in the low-risk group plateau, while the high-risk group exhibits a slower rate of increase, with levels remaining noticeably lower after age 14 (**Figure 2b**).

### Sensitivity analyses

In the sensitivity analysis, the results were not substantially altered when we repeated the analysis by 1) further adjusting for variables (such as household income, parental education level, **Supplementary Table S3**), and **Supplementary Table S2**) categorizing IGF-1 and IGFBP-3 into tertiles (**Supplementary Table S4**).

## Discussion

In this cross-sectional study, we identified that high levels of IGF-1, IGFBP-3, and their ratio were associated with metabolic abnormalities among children and adolescents. Additionally, within the observed age range, the levels of IGF-1 and IGFBP-3 were higher in the low-risk group compared to the high-risk group.

IGF-1 is an important biomarker for the assessment of growth abnormalities (27–29) such as growth retardation or dwarfism (30) in children and adolescent populations. Notably, recent population studies have shown that IGF-1 levels are lower when individuals suffer from conditions such as obesity (31), lipid metabolism disorders (32), hypertension (33), and metabolic syndrome (34). Meanwhile, some studies have shown that low levels of IGF-1 are associated with the components of metabolic syndrome, including elevated triglycerides (35) and reduced HDL cholesterol levels (34), which is consistent with our findings. Most of these studies have focused on older people or people suffering from other diseases. To our knowledge, only a few studies have evaluated the association of IGF-1 and IGFBP3 with metabolic abnormalities in children and adolescents, finding an association of lower IGF-1 and IGFBP-3 with metabolic abnormalities (36) and that improving the status of obesity and insulin resistance in children and adolescents may further augment the functionality of the IGF-1 axis (37). Our study found that high IGF-1 and IGBP-3 were related to metabolic abnormalities in children and adolescent populations.

Several mechanisms explain the association of IGF-1 and IGFBP-3 with metabolic abnormalities. Firstly, In the body, insulin not only regulates energy metabolism by promoting peripheral glucose uptake but also acts at high concentrations in the portal vein on hepatocytes to upregulate GH receptor expression and enhance GH-driven IGF-1 synthesis (38). Most synthesized IGF-1 circulates bound in a ternary complex with IGFBP-3 and the acid-labile subunit, which prolongs its half-life and regulates its delivery to tissues (39). IGFBP-3 levels are co-regulated by GH and insulin, allowing it to both stabilize IGF-1 and, via IGF-independent mechanisms—including inhibition of IRS-1/IR-β tyrosine phosphorylation, reduction of Akt phosphorylation and GLUT-4 translocation, and downregulation of adiponectin—to antagonize insulin signaling in peripheral tissues and promote insulin resistance (40). Second, IGF-1 affects lipid metabolism and fat storage in the body by regulating the expression of key enzymes involved in lipid metabolism such as fatty acid synthase (FAS) (41) and lipoprotein lipase (LPL) (42). In *in vitro* studies, IGFBP-3 significantly inhibited insulin-stimulated glucose transport and the expression of adiponectin in adipocytes, showing an insulin-antagonistic effect. Adiponectin is a hormone related to insulin sensitivity, and inhibition of its expression with IGFBP-3 may further exacerbate insulin resistance (43). In animal study, managing IGFBP-3 expression influences the growth and differentiation of brown preadipocytes, suggesting that IGFBP-3 plays a key role in regulating brown adipocyte fate (44). Thirdly, IGF-1 stimulates the endothelial cells to produce nitric oxide (NO), which can improve vasodilation and decrease vascular stiffness (45). Finally, IGF-1 and IGFBP-3 reduce TNF-α, IL-6, and other inflammatory indicators, which are critical for decreasing chronic low-grade inflammation in the progression of metabolic syndrome (46).Although our research lacked formal assessments of mood disorders or seasonal hormone measurements, prior studies have documented pronounced seasonal fluctuations in GH secretion (47), underscoring the importance of temporal hormonal dynamics in endocrine–metabolic research.

The strength of this study included exploring the role of IGF-1 and IGFBP-3 in childhood metabolism. However, some limitations should be acknowledged. Firstly, since the cross-sectional design of our study, we cannot infer causation. Secondly, given that the study population was from China, caution should be taken in extrapolating the results to other racial or ethnic groups. Also, Our cohort did not collect Tanner staging or other formal pubertal assessments; therefore, we were unable to perform analyses stratified by pubertal status. Finally, although we adjusted for several confounders, we did not incorporate other confounders such as genetic variation that potentially affects the association.

## Conclusion

In conclusion, this study found that association of higher IGF-1 and IGFBP-3 with a lower risk of metabolic abnormalities in children and adolescents. Our findings highlight that maintaining IGF-1 and IGBP-3 homeostasis is negatively associated with the risk of metabolic abnormalities in children and adolescents. Future studies are needed to explore the mechanisms as well as to further verify this association through cohort studies.

## Data Availability

The raw data supporting the conclusions of this article will be made available by the authors, without undue reservation.
