# Supplementary material for: Association of IGF-1 and IGFBP-3 with metabolic abnormalities among children and adolescents
Source: Front Endocrinol (Lausanne). 2025 Jun 5;16:1579107. doi: 10.3389/fendo.2025.1579107 (PMC12176600; doi:10.3389/fendo.2025.1579107)
Supplement: Supplementary file 1 [file DataSheet1.docx]

**Table S1.**The association of IGF-1 and IGFBP-3 with metabolic abnormalities among children and adolescents stratified by sex

|  | Male | | Female | |
| --- | --- | --- | --- | --- |
|  | OR | P | OR | P |
| **IGF-1(ng/ml)** | | | | |
| Model |  |  |  |  |
| Q1 | Reference | | Reference | |
| Q2 | 0.36(0.12,1.03) | 0.361 | 0.47(0.16,1.34) | 0.162 |
| Q3 | 0.17(0.05,0.50) | 0.002 | 0.44(0.15,1.25) | 0.126 |
| Q4 | 0.12(0.04,0.39) | <0.001 | 0.26(0.09,0.75) | 0.015 |
| **IGFBP-3(μg/ml)** | | | | |
| Model |  |  |  |  |
| Q1 | Reference | | Reference | |
| Q2 | 0.62(0.24,1.57) | 0.315 | 0.32(0.11,0.94) | 0.042 |
| Q3 | 0.25(0.08,0.69) | 0.001 | 0.49(0.17,1.33) | 0.162 |
| Q4 | 0.33(0.11,0.93) | 0.039 | 0.25(0.08,0.72) | 0.013 |

IGF-1: Q1:< 214.8 ng/ml; Q2: 214.8-433.5 ng/ml; Q3: 433.5-983.00 ng/ml; Q4: ≥ 983.00 ng/ml.

IGFBP-3: Q1: < 4.84 μg/ml; Q2: 4.84-5.76 μg/ml; Q3: 5.76-12.00 μg/ml; Q4: ≥ 12.00 μg/ml.

Model adjusted for age, BMI, WHR, ALT, AST, TBIL, DBIL, TG, HOMA, HDL-C and LDL-C.

**Table S2.**The association of IGF-1 and IGFBP-3 with metabolic abnormalities among children and adolescents stratified by age

|  | 6-12 | | 13-17 | |
| --- | --- | --- | --- | --- |
|  | OR | P | OR | P |
| **IGF-1(ng/ml)** | | | | |
| Model |  |  |  |  |
| Q1 | Reference | | Reference | |
| Q2 | 0.60(0.28,1.26) | 0.179 | 0.08(0.01,1.05) | 0.059 |
| Q3 | 0.35(0.15,0.79) | 0.012 | 0.05(0.01,0.57) | 0.023 |
| Q4 | 0.29(0.12,0.68) | 0.005 | 0.02(0.01,0.29) | 0.006 |
| **IGFBP-3(μg/ml)** | | | | |
| Model |  |  |  |  |
| Q1 | Reference | | Reference | |
| Q2 | 0.68(0.32,1.42) | 0.310 | 0.04(0.01,0.42) | 0.018 |
| Q3 | 0.40(0.18,0.83) | 0.016 | 0.08(0.01,0.85) | 0.063 |
| Q4 | 0.63(0.28,1.38) | 0.252 | 0.01(0.01,0.11) | 0.002 |

IGF-1: Q1:< 214.8 ng/ml; Q2: 214.8-433.5 ng/ml; Q3: 433.5-983.00 ng/ml; Q4: ≥ 983.00 ng/ml.

IGFBP-3: Q1: < 4.84 μg/ml; Q2: 4.84-5.76 μg/ml; Q3: 5.76-12.00 μg/ml; Q4: ≥ 12.00 μg/ml.

Model adjusted for sex, BMI, WHR, ALT, AST, TBIL, DBIL, TG, HOMA, HDL-C and LDL-C.

**Table S3.** Sensitivity Analysis of the association of IGF-1 and IGFBP-3 with metabolic risk in children and adolescents.

|  | **OR (95% CI)** | ***P*-value** |
| --- | --- | --- |
| **IGF-1 (ng/ml)** | | |
| Model 1 | | |
| Q1 | Reference |  |
| Q2 | 0.61 (0.40, 0.91) | 0.016 |
| Q3 | 0.40 (0.26, 0.61) | < 0.001 |
| Model 2 | | |
| Q1 | Reference |  |
| Q2 | 0.42 (0.23, 0.78) | 0.006 |
| Q3 | 0.24 (0.12, 0.46) | < 0.001 |
| Model 3 | | |
| Q1 | Reference |  |
| Q2 | 0.41 (0.22, 0.77) | 0.005 |
| Q3 | 0.25 (0.13, 0.48) | 0.003 |
| **IGFBP-3(μg/ml)** | | |
| Model 1 | | |
| Q1 | Reference |  |
| Q2 | 0.55 (0.36, 0.83) | 0.005 |
| Q3 | 0.52 (0.34, 0.79) | 0.002 |
| Model 2 |  |  |
| Q1 | Reference |  |
| Q2 | 0.43 (0.23, 0.78) | 0.006 |
| Q3 | 0.34 (0.18, 0.63) | <0.001 |
| Model 3 | | |
| Q1 | Reference |  |
| Q2 | 0.43 (0.23, 0.80) | 0.007 |
| Q3 | 0.35 (0.19, 0.65) | 0.001 |

IGF-1: Q1:< 248 ng/ml; Q2: 248-393 ng/ml; Q3: ≥393 ng/ml.

IGFBP-3: Q1: < 5.08 μg/ml; Q2: 5.08-6.20 μg/ml; Q4: ≥6.20 μg/ml.

Model 1 is the initial model.

Model 2 adjusted for gender, age, BMI, Waist-Hip Ratio (WHR), ALT, AST, TBIL, DBIL, TG, HOMA, HDL, LDL.

Model 3 was further adjusted for family income, father education and mother education.

**Table S4.** Sensitivity Analysis of the association between IGF-1/IGFBP-3 ratio and metabolic risk in children and adolescents

|  | OR (95% CI) | *P*-value |
| --- | --- | --- |
| **IGF-1/IGFBP-3** | | |
| Model 1 | | |
| Q1(N=199) | Reference |  |
| Q2(N=195) | 0.51 (0.33, 0.77) | 0.001 |
| Q3(N=194) | 0.52 (0.34, 0.78) | 0.002 |
| Model 2 | | |
| Q1 | Reference |  |
| Q2 | 0.58 (0.23, 0.78) | 0.071 |
| Q3 | 0.40 (0.12, 0.46) | 0.003 |
| Model 3 | | |
| Q1 | Reference |  |
| Q2 | 0.59 (0.32, 1.07) | 0.005 |
| Q3 | 0.39 (0.13, 0.48) | 0.003 |

IGF-1/IGFBP-3 ratio: Q1:<47.07; Q2: 47.07-67.30; Q4: ≥67.30.

Model 1 is the initial model.

Model 2 adjusted for gender, age, BMI, Waist-Hip Ratio (WHR), ALT, AST, TBIL, DBIL, TG, HOMA, HDL, LDL.

Model 3 was further adjusted for family income, father education and mother education.
